# Iron status in Swiss adolescents with paediatric major depressive disorder and healthy controls: a matched case–control study

**DOI:** 10.1007/s00394-023-03313-7

**Published:** 2024-01-24

**Authors:** Ester Osuna, Jeannine Baumgartner, Olivia Wunderlin, Sophie Emery, Mona Albermann, Noemi Baumgartner, Klaus Schmeck, Susanne Walitza, Michael Strumberger, Martin Hersberger, Michael B. Zimmermann, Isabelle Häberling, Gregor Berger, Isabelle Herter-Aeberli, the Omega-3 Study Team, Mona Albermann, Kristin Nalani, Oliver Pick, Alain Di Gallo, Michael Strumberger, Brigitte Contin, Stefan Müller, Silke Bachmann, Lars Wöckel, Simone Heitzer, Bruno Rhiner, Amir Yamini, Suzanne Erb, Michael Schmid, Ulrich Müller-Knapp, Ioannis Christodoulakis, Burkhardt Seifert, Renate Drechsler, Edna Grünblatt, Martin Hersberger, Martin Volleberg, Ivan Hartling, Akash Chakravarty, Romuald Brunner, Jürgen Drewe, Julia Braun, Jenny Peterson

**Affiliations:** 1 Laboratory of Human Nutrition, Institute of Food, Nutrition and Health, ETH Zürich, Zurich, Switzerland; 2 Department of Nutritional Sciences, King’s College London, London, UK; 3 Department of Child and Adolescent Psychiatry and Psychotherapy, Psychiatric Hospital, University of Zurich, Zurich, Switzerland; 4 Psychiatry St. Gallen, Wil SG, Switzerland; 5 Research Department of Child and Adolescent Psychiatry, Psychiatric University Hospitals Basel, University of Basel, Basel, Switzerland; 6 Division of Clinical Chemistry and Biochemistry, University Children’s Hospital Zurich, University of Zurich, Zurich, Switzerland; 7 Department of Clinical Research, Medical Faculty, University of Basel, Basel, Switzerland; 8 Laboratory of Nutrition and Metabolic Epigenetics, Institute of Food, Nutrition, and Health, ETH Zurich, Zurich, Switzerland

**Keywords:** Iron status, Iron deficiency, Depression, Major depressive disorder, Adolescents, Inflammation, iFABP, Case–control study, Paediatric major depressive disorder

## Abstract

**Purpose:**

Depression is associated with low-grade systemic inflammation and impaired intestinal function, both of which may reduce dietary iron absorption. Low iron status has been associated with depression in adults and adolescents. In Swiss adolescents, we determined the associations between paediatric major depressive disorder (pMDD), inflammation, intestinal permeability and iron status.

**Methods:**

This is a matched case–control study in 95 adolescents with diagnosed pMDD and 95 healthy controls aged 13–17 years. We assessed depression severity using the Children’s Depression Rating Scale-Revised. We measured iron status (serum ferritin (SF) and soluble transferrin receptor (sTfR)), inflammation (C-reactive protein (CRP) and alpha-1-acid-glycoprotein (AGP)), and intestinal permeability (intestinal fatty acid binding protein (I-FABP)). We assessed history of ID diagnosis and treatment with a self-reported questionnaire.

**Results:**

SF concentrations did not differ between adolescents with pMDD (median (IQR) SF: 31.2 (20.2, 57.0) μg/L) and controls (32.5 (22.6, 48.3) μg/L, *p* = 0.4). sTfR was lower among cases than controls (4.50 (4.00, 5.50) mg/L vs 5.20 (4.75, 6.10) mg/L, *p* < 0.001). CRP, AGP and I-FABP were higher among cases than controls (CRP: 0.16 (0.03, 0.43) mg/L vs 0.04 (0.02, 0.30) mg/L, *p* = 0.003; AGP: 0.57 (0.44, 0.70) g/L vs 0.52 (0.41, 0.67) g/L, *p* = 0.024); I-FABP: 307 (17, 515) pg/mL vs 232 (163, 357) pg/mL, *p* = 0.047). Of cases, 44% reported having a history of ID diagnosis compared to 26% among controls (*p* = 0.020). Finally, 28% of cases had iron treatment at/close to study inclusion compared to 14% among controls.

**Conclusion:**

Cases had significantly higher systemic inflammation and intestinal permeability than controls but did not have lower iron status. Whether this is related to the higher rate of ID diagnosis and iron treatment in adolescents with depression is uncertain.

**Supplementary Information:**

The online version contains supplementary material available at 10.1007/s00394-023-03313-7.

## Introduction

Depression, a leading cause of disability [1], affects an estimated 300 million people worldwide [2]. Paediatric major depressive disorder (pMDD) is one of the most common psychiatric disorders during childhood and adolescence [3]. It is estimated that 11% of adolescents have had an episode of pMDD during their lifetime [4]. Early onset of depression is a risk factor for chronic and recurrent forms of depression in adulthood [5]. Furthermore, pMDD is associated with poor educational, work, and social functioning as well as an increased rate of smoking, substance abuse, eating disorders, and obesity [6]. However, pMDD often remains undiagnosed and, therefore, untreated [7]. Furthermore, the aetiology of pMDD is poorly understood and believed to be multifactorial [8].

Depression has been associated with inflammation and an altered immune response [9]. Inflammation can decrease intestinal iron absorption by upregulating the synthesis of the iron-regulating hormone hepcidin [10]. Furthermore, emerging evidence indicates that depression is associated with increased intestinal permeability [11–13], which may be caused by gut microbiota dysbiosis and may trigger low-grade systemic inflammation. This low-grade systemic inflammation may in turn contribute to decreased intestinal iron absorption as a result of further upregulation of hepcidin. Thus, depression-associated inflammation and increased intestinal permeability may contribute to the development of iron deficiency (ID) in depressed individuals, which could potentially aggravate disease severity.

In children and adolescents, ID has been associated with poor school performance, decreased cognitive abilities, and behavioural problems [14, 15]. Iron, a key co-factor in the electron transfer reaction of cellular respiration and component of the oxygen transporter haemoglobin [16], is known to play a crucial role in neurodevelopment by being responsible for gene regulation, and regulation of cell growth and differentiation [17, 18]. Furthermore, iron is an important co-factor for the enzymes responsible for myelination of neurons and for the synthesis of dopamine and serotonin [19]. Thus, considering that neurodevelopmental processes such as myelination and synaptogenesis (as well as synaptic pruning) are ongoing processes during adolescence, ID could be involved in the aetiology of depression by contributing to structural and functional changes in brain architecture driven by mitochondrial dysfunction [20], as well as alterations in monoaminergic neurotransmission, which have been linked with depression [21–24].

Previous studies did associate low iron status with depression [25–29]. The interpretation of the most used iron status biomarker serum ferritin (SF) however, is difficult in populations with widespread inflammation such as depressive disorders [9]. SF is an acute phase reactant, responding to inflammation by rapid upregulation independent of iron stores [30]. Thus, inflammatory markers such as C-reactive protein (CRP) and alpha-1-acid-glycoprotein (AGP) need to be considered when evaluating iron status in depressed individuals.

In Switzerland, surveys suggest the prevalence of depressive symptoms to be around 10% [31]. Data on iron status of Swiss adolescents are scarce. Recently, daily intake of iron in young Swiss adults (18–34 years) was determined in a dietary survey and was estimated to be 10.3 mg/day for men and 9.0 mg/day for women [32]. These intakes are below the European Food Safety Authority (EFSA) population reference intake values for males aged 12–17 years of 11 mg/day and for 12–17-year-old females of 13 mg/day [33].

Thus, the aim of this study was to determine the association between iron status and pMDD in Swiss adolescents. Considering the potential bi-directional relationship between depression and ID, we further explored the associations of systemic inflammation and intestinal permeability with depression. We hypothesised that adolescents with pMDD would have a lower iron status compared to adolescents without pMDD. We further hypothesised that adolescents with pMDD would have increased levels of inflammation and impaired gut permeability, and that biomarkers of inflammation and gut permeability are correlated with biomarkers of iron status. We further assessed the history of ID diagnosis and treatment, considering that adolescents seeking medical care with depressive symptoms might get diagnosed with ID as part of routine care.

## Participants and methods

### Study design

This study is an observational matched case–control study in 13–17-year-old adolescents with diagnosed pMDD and healthy controls. The adolescents were matched in a 1:1 ratio according to sex, age group (13 to < 16 and 16 to < 18 years) and education level. The sample size calculation for this study was performed with G*Power V3.1.9.2. A logistic model, where the Children’s Depression Rating Scale-Revised (CDRS-R) score for depression severity was coded as a dichotomous variable in a model with 10 covariates (residual *R*^2^ = 0.2) and one standard deviation (SD) increase of the continuous predictor generated an odds ratio (OR) of 1.5 and 2, was used as the basis for a power calculation. According to these power calculations, to detect medium to large effect sizes for a type-I error of 5% (alpha = 0.05) a sample size of 200 individuals with a 1:1 matching case–control ratio was sufficient (power > 80%, beta ≥ 20%). Up to a drop-out rate of 10%, these results seemed robust. To have a balanced sample, we aimed to include 102 cases and 102 controls, with equal representation of sex, age groups, and education level in cases and controls. In the first age group (13 to < 16 years), the aim was to include 50 adolescents (25 females and 25 males) for each the cases and the controls. In the second age group (16 to < 18 years), the aim was to recruit 52 adolescents (26 females and 26 males) for both, the cases and the controls. In the younger age group, all adolescents attended lower secondary school level (mandatory school years in Switzerland). For the older age group, adolescents were further matched based on their higher secondary school educational level (*n* = 26 from each level): (1) vocational education (apprenticeship), and (2) baccalaureate/vocational baccalaureate. In this case–control study, the recruitment of controls followed the mentioned recruitment strategy and the cases were then randomly selected to match the controls.

The ethics committee of the Canton of Zurich approved this study (BASEC-Nr. 2019-00717) and the study was registered at www.ClinicalTrials.gov (NCT04158869). The study was approved as an add-on study to the investigator-initiated clinical trial (SNSF 33IC30_166826, BASEC-Nr. 2016-02116). Written informed consent was obtained from all the caregivers and adolescents ≥ 14 years of age, and adolescents < 14 years of age gave their oral assent before any research-related assessments were conducted.

### Participants and procedures

#### Control group

The Laboratory of Human Nutrition at ETH Zurich, Switzerland, recruited the healthy controls for this study. From the canton of Zurich and surrounding German-speaking cantons of Switzerland, healthy female and male controls were recruited from September 2019 until December 2020. Schools, leisure time clubs, and social media were sites for recruiting the controls. Inclusion criteria for controls were no present nor past primary diagnosed psychiatric disorder according to the Mini-International Neuropsychiatric Interview for Children and Adolescents (M.I.N.I. KID) [34]; age of 13 to < 18 years; and no use of chronic medication. Adolescents were not eligible as controls if they reported pre-existing neurological or medical conditions likely to be a risk factor for developing depressive symptoms; if they took n-3 PUFA supplements (providing > 600 mg combined EPA/DHA) for more than 4 weeks within the last 6 months; or if they were unable to follow the study procedures, for example, due to language barriers. Once consent was given and the participants enrolled into the study, they electronically completed questionnaires on REDCap® (Research Electronic Data Capture) within 2 weeks prior to the physical data assessment at ETH Zurich.

### pMDD group

The cases in this study were randomly selected from the participants of the *Omega-3 Fatty Acids as treatment for Paediatric Major Depressive Disorder Trial (Omega-3 pMDD)* under the lead of the Psychiatric University Hospital Zurich to match the controls. The Omega-3 pMDD protocol has been published before [35]. The recruitment for the omega-3 pMDD study took place at seven different in- and outpatient service centres in five German-speaking cantons of Switzerland from May 2017 until June 2021. The information about the study reached the adolescents either via their clinician or via posters and flyers in one of the participating centres. When seeing the study on their own initiative, individuals contacted the study team themselves without the involvement of a clinician. Inclusion criteria for teenagers were age of 13 to < 18 years; and a main diagnosis of MDD according to DSM-IV criteria [36] of at least moderate severity defined by a CDRS-R total score of ≥ 40 [37]. Adolescents were not eligible if they had a lifetime diagnosis of schizophrenia, bipolar affective disorder, substance use dependency, mental retardation, or pervasive development disorder; or if they fulfilled the diagnostic criteria for an eating disorder within the last 6 months. In addition, cases were not eligible if they had pre-existing neurological or medical conditions, which were likely to cause their depressive symptoms. Furthermore, if cases were taking n-3 PUFA supplements (> 600 mg combined EPA/DHA) within the last 6 months; or if they or their families were unable to follow the study procedures, for example, due to language barriers, they were not eligible for the study. After consenting to the study, the screening interview was conducted with the adolescents and a parent separately. In this screening interview, inclusion and exclusion criteria were assessed using the Kiddie-Schedule for Affective Disorders and Schizophrenia (K-SADS) [38] for assessing the presence of MDD and the CDRS-R for assessing the severity of the depression. For this case–control study, only data (biological samples and CDRS-R scores) from the baseline assessment before randomisation was used.

### Data collection

The study team made every possible effort to align the study procedures between controls and cases as much as possible.

### Assessment of anthropometry and socio-demographic information

For the cases and the controls, weight (to the nearest 0.1 kg) and height (to the nearest 0.5 cm) were measured. Thereof, the body mass index (BMI) was calculated as body weight in kilograms (kg) over the person’s height in metres (m) squared (BMI = kg/m^2^). Further, BMI-for-age z-scores were calculated with the R package “anthroplus” which is provided by the WHO and uses the children’s and adolescent’s growth reference data [39]. BMI data were then age-dependently categorised into four categories: underweight, normal weight, overweight and obese according to the WHO’s reference values [40]. In adolescents, a *z*-score < -1 coincides with adult underweight (BMI < 18), while a *z*-score >  + 1 coincides with adult overweight (BMI ≥ 25) and a *z*-score >  + 2 with adult obesity (BMI ≥ 30). Socio-economic and demographic data were assessed by self-reporting questionnaires which the participants were asked to fill out together with one parent.

### Assessment of depression severity

We used the CDRS-R to assess adolescents’ presence and severity of depression [37]. The CDRS-R, a semi-structured clinical interview which takes 15–20 min to administer, is one of the most frequently used rating scales for measuring the severity of depression and the change in depressive symptoms in children and adolescents with depression [41]. The validity of the scale has been established for children [42] and adolescents [41]. The interview allows a comprehensive assessment by providing the possibility of conducting it with the child, the parents, and/or teacher. It covers 17 depressive symptom areas which are rated on 5- to 7-point Likert rating scales. The depressive symptom domains are aligned with the DSM-IV criteria for childhood depression [43] and include sleep disturbance, excessive fatigue, suicidal ideation, and social withdrawal. Participants are asked about information on 14 items. Further, three non-verbal symptoms, such as depressed facial affect, are rated only by the interviewer. The interviewers were trained to conduct the interview. Based on the individual ratings, a total score is calculated, ranging between 17 and 113. For this study, scores from the interview conducted with the adolescent were used and a score of ≥ 40 was used as cutoff for pMDD [44].

### Questionnaire on history of iron diagnosis and treatment

Participants were asked to fill out a questionnaire on the history of ID diagnosis and ID treatment in the form of oral iron supplementation or intravenous infusion. This questionnaire assessed the lifetime history of ID diagnosis and time point of ID treatment. To control for the confounding effect of iron treatment on iron status in this study, we defined treatment at or close to study inclusion as up to 1 year prior to inclusion, based on the estimated effect duration of iron supplementation of 1 year [45]. For cases and controls, this questionnaire was introduced after recruitment and data assessment had already started. Therefore, for some participants, this questionnaire was answered retrospectively to inclusion and completion of the study.

### Biochemical analysis

Blood samples were collected into EDTA-coated tubes and serum tubes (BD Vacutainer). Haemoglobin (Hb) was measured in whole blood using a Sysmex XE_5000 analyzer (Sysmex Corporation). Serum tubes were let to stand for 60 min to allow clotting. Afterwards, the serum tubes were centrifuged, and the serum was then stored at −80 °C until further analysis.

We analysed iron status by measuring SF and soluble Transferrin Receptor (sTfR) and inflammation by measuring CRP and AGP using a multiplex immunoassay [46]. SF values were adjusted for inflammation using the BRINDA method using the R package “BRINDA”, applying “other” population groups as reference [30]. Iron deficiency was defined as inflammation-adjusted SF values < 15 µg/L and/or sTfR > 8.3 mg/L. Anaemia was defined according to the age- and sex-dependent WHO cutoff values for Hb [47, 48]. For all female participants as well as male participants between 12 and 14 years of age, anaemia was defined as Hb < 120 g/L. For male participants 15 years of age and above, anaemia was defined as Hb < 130 g/L. Iron deficiency anaemia (IDA) was defined as the combination of ID with anaemia. CRP > 5 mg/L and/or AGP > 1 g/L were used to define inflammation [49]. I-FABP concentrations were measured using a commercially available Enzyme-Linked Immunosorbent Assay (ELISA) (Hycult Biotech, Uden, The Netherlands).

### Data management and statistical methods

For the controls, the data capture was done either electronically using REDCap® or on paper and later entered to the REDCap® system. When captured on paper, the data were entered by the assessing person and later double checked for entry errors by a second member of the study team. REDCap® is an electronic data capture tool hosted at ETH Zurich and provides a secure, web-based software platform designed for supporting data capture in research studies [50, 51]. For the cases, data were assessed on paper and then entered to the electronical data capture tool secuTRIAL by two individual persons. Afterwards, every data entry was checked by a third person for entry errors. Once cases and controls were matched, study data were managed using REDCap®.

Data processing and statistical analysis of data were performed using R Version 3.6.0 [52]. To test for outliers and normality, Q–Q plots, histograms, and the Shapiro–Wilk test were used. Normally distributed data and non-normally distributed data were expressed as mean (± SD) and as medians (Interquartile range, IQR), respectively. For comparing not normally distributed continuous data between cases and controls, Wilcoxon rank sum test was applied, and t-test for normally distributed data. Chi-square tests were applied to test for differences between cases and controls when the expected cell count was ≥ 5, and Fisher’s exact test when the expected cell count was < 5. For producing tables and calculating these differences, the R package “gtsummary” was used. This R package has been shown to produce reproducible summary tables within R [53]. To determine correlations between two non-normally distributed variables, Spearman’s correlation coefficient was calculated. Further, to assess associations of different iron status parameters with depression (CDRS score ≥ 40), multivariate logistic regression analysis was applied. In these models, we used the matching criteria sex, age, and education level, as well as BMI-for-age z-scores, antidepressant use, and AGP as covariates.

## Results

**Fig. 1:**
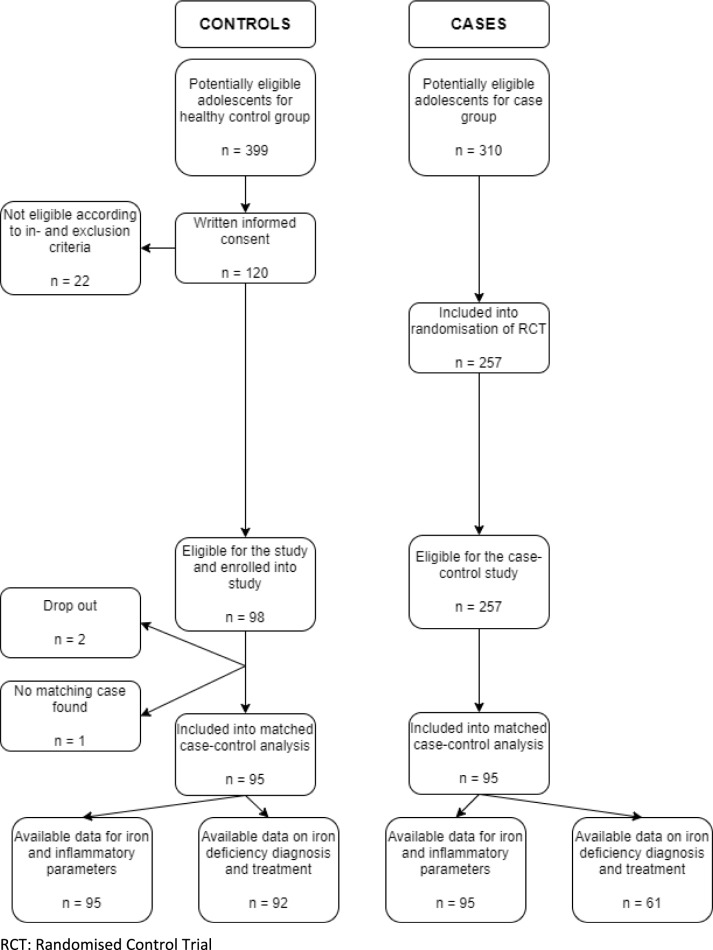
Flow chart of adolescents with and without diagnosed paediatric major depressive disorder included in the analysis of this case–control study. *RCT* randomised control trial

A total of 98 controls were enrolled into this case–control study after the recruitment process. Thereof, two individuals dropped out, either by not providing a blood sample or voluntarily after the screening interview. A total of 257 participants were randomised to one of the treatment arms of the Omega-3 pMDD study and were, therefore, eligible as cases for this case–control study. For one control, no match within the cases could be found. Consequently, a total of 95 controls were matched to 95 cases (total *n* = 190) according to sex, age group and education level. A detailed overview of the study inclusion process is given in Fig. 1. For four adolescent pairs in the age group of 16 to < 18 years of age, the matching was done according to sex and age group since there was no match by education level.

Table 1 provides a detailed overview of the study participants’ characteristics. The successful matching between cases and controls is demonstrated by no significant difference in age, sex, and education level. Furthermore, BMI-for-age *z*-scores were comparable between the groups. CDRS-R scores were significantly higher among the cases compared to the controls (*p* < 0.001). A slightly higher number of adolescents among the cases were of East-Asian descent compared to controls (*p* = 0.051). Among the participants, 22% of cases had a recurrent episode of depression. The use of antidepressants among cases at study inclusion was 38%. Finally, the use of antidepressant drugs at study inclusion correlated with the recurrence of episodes (*r*_*s*_ = 0.29, *p* < 0.001).

**Table 1:** Characteristics of Swiss adolescents with (*n* = 95) and without (*n* = 95) paediatric major depressive disorder (pMDD)

	Overall	Cases	Controls	*p*-Value
Age	16.1 (14.9, 17.1)	16.1 (14.9, 17.2)	16.0 (14.9, 17.1)	0.8^b^
Sex				> 0.9^c^
Female	110 (58%)	55 (58%)	55 (58%)	
Male	80 (42%)	40 (42%)	40 (42%)	
CDRS score	36 (18, 56)	56 (50, 62)	18 (17, 20)	** < 0.001**^b^
BMI-for-age *z*-score^a^	0.20 ± 1.03	0.25 ± 1.05	0.16 ± 1.02	0.4^b^
BMI^a^				0.5^d^
Underweight	7 (4%)	2 (2%)	5 (5%)	
Normal weight	138 (76%)	65 (75%)	73 (77%)	
Overweight	20 (11%)	12 (14%)	8 (8%)	
Obese	17 (9%)	8 (9%)	9 (10%)	
Ethnicity				0.051^d^
European	174 (92%)	87 (92%)	87 (92%)	
East-Asian	5 (3%)	5 (5%)	0 (0%)	
Indian-Asian	2 (1%)	0 (0%)	2 (2%)	
Middle East	2 (1%)	1 (1%)	1 (1%)	
Not declared	7 (4%)	2 (2%)	5 (5%)	
Swiss education levels				0.8^c^
Lower secondary school (mandatory school)	104 (55%)	54 (57%)	50 (53%)	
Upper secondary school				
Vocational education	28 (15%)	14 (15%)	14 (15%)	
Baccalaureate/vocational baccalaureate	58 (31%)	27 (28%)	31 (33%)	
Participants using antidepressants at study inclusion	36 (19%)	36 (38%)	0 (0%)	**< 0.001**^c^
Participants with recurrent episode at study inclusion	22 (12%)	22 (23%)	0 (0%)	**< 0.001**^c^
Participants with recurrent episode AND using antidepressants	11 (6%)	11 (12%)	0 (0%)	**< 0.001**^c^

Median (IQR); mean ± SD, *n* (%); *p*-values in bold were statistically significant

*CDRS* children’s depression rating scale; *IQR* interquartile range; *SD* standard deviation

^a^Data on Body Mass Index (BMI) was not available for all participants (*n*_cases_ = 87; *n*_controls_ = 95), BMI-for-age *z*-scores and BMI categories were defined according to WHO reference data

^b^Wilcoxon rank sum test

^c^Pearson’s Chi-squared test

^d^Fisher’s exact test

A detailed description of the iron and inflammatory parameters is given in Table 2. There was no significant difference in SF concentrations between cases and controls (median (IQR): 37.2 (20.2, 57.0) μg/L; 32.5 (22.6, 48.3) μg/L, *p* = 0.4), also when adjusting for inflammation or investigating differences by sex (*p* = 0.7 in females and *p* = 0.3 in males, shown in Supplementary Table 1). However, in both cases and controls, we observed lower SF concentrations in females compared to males (cases: 31.8 (18.0, 46.9) μg/L vs 47.2 (33.8, 64.4) μg/L, *p* = 0.007 and controls: 29.2 (16.3, 43.6) μg/L vs 42.1 (26.0, 53.2) μg/L, *p* = 0.012). sTfR concentrations were lower among cases compared to controls (4.50 (4.00, 5.50) mg/L; 5.20 (4.75, 6.10) mg/L, *p* < 0.001); this was seen in both females (*p* = 0.006) and males (*p* < 0.001). Furthermore, there was a trend for higher body iron stores (BIS) among the cases compared to the controls (mean ± SD: cases: 5.73 ± 3.31 mg/kg; controls: 5.00 ± 2.92 mg/kg, *p* = 0.083). The prevalence of ID (adjusted SF values < 15 µg/L and/or sTfR > 8.3 mg/L) among cases (19%) and controls (18%) was comparable (*p* = 0.7). The prevalence of ID was higher in females (26%) than males (11%). Furthermore, there were no differences in ID prevalence between cases and controls among females and males separately (females: 14 (26%) vs 14 (26%) and males: 5 (13%) vs 4 (10%)).

**Table 2:** Summary of iron and inflammatory parameters for adolescents with (*n* = 95) and without (*n* = 95) paediatric major depressive disorder (pMDD)

Characteristic	Overall	Cases	Controls	*p*-Value
SF (μg/L)	35.6 (21.4, 52.8)	37.2 (20.2, 57.0)	32.5 (22.6, 48.3)	0.4^2^
Adjusted SF (μg/L)	32.5 (20.0, 46.2)	32.7 (19.1, 53.1)	30.8 (21.1, 45.0)	0.6^2^
sTfR (mg/L)	4.99 (4.25, 5.76)	4.50 (4.00, 5.50)	5.20 (4.75, 6.10)	** < 0.001**^g^
Adjusted sTfR (mg/L)	4.72 (4.05, 5.46)	4.20 (3.79, 5.00)	4.94 (4.48, 5.92)	** < 0.001**^g^
Body iron stores (BIS) (mg/kg)	5.36 ± 3.13	5.73 ± 3.31	5.00 ± 2.92	0.083^g^
Haemoglobin (g/L)^a^	140.7 ± 12.5	140.6 ± 13.8	140.8 ± 11.2	0.8^g^
CRP (mg/L)	0.10 (0.02, 0.35)	0.16 (0.03, 0.43)	0.04 (0.02, 0.30)	**0.003**^g^
AGP (g/L)	0.57 (0.44, 0.70)	0.60 (0.47, 0.74)	0.52 (0.41, 0.67)	**0.024**^g^
I-FABP (pg/mL)	267 (168, 448)	307 (17, 515)	232 (163, 357)	**0.047**^g^
Iron deficiency (ID)^b^	37 (19%)	19 (20%)	18 (19%)	0.9^h^
Low iron stores^c^	35 (18%)	18 (19%)	17 (18%)	0.9^h^
Anaemia (A)^a,d^				0.3^i^
No A	179 (96%)	86 (93%)	93 (98%)	
Mild A	6 (3%)	4 (4%)	2 (2%)	
Moderate A	2 (1%)	2 (2%)	0 (0%)	
Iron deficiency anaemia (IDA)^a^	5 (3%)	4 (4%)	1 (1%)	0.2^i^
Iron deficiency erythropoiesis (IDE)^e^	5 (3%)	2 (2%)	3 (3%)	> 0.9
Elevated inflammatory status^f^	15 (8%)	9 (10%)	6 (6%)	0.4^h^

Median (interquartile range); mean ± SD; *n* (%); *p*-values in bold were statistically significant

*AGP* alpha-1-acid-glycoprotein; *CRP* C-reactive protein; *I-FABP* intestinal fatty acid binding protein; *SD* standard deviation; *SF* serum ferritin; *sTfR* soluble transferrin receptor

^a^Data on haemoglobin were not available for all participants (*n*_cases_ = 92; *n*_coltrols_ = 95)

^b^ID was defined as adjusted SF < 15 μg/L and/or elevated sTfR > 8.3 mg/L

^c^Low iron stores defined as adjusted SF < 15 μg/L

^d^Anaemia was defined according to age and sex specific WHO cut-off values (displayed in Sect. 2.3.4)

^e^IDE was defined as elevated adjusted sTFR > 8.3 mg/L

^f^Inflammation was defined as CRP > 5 mg/L and/or AGP > 1 g/L

^g^Wilcoxon rank sum test

^h^Pearson’s Chi-squared test

^i^Fisher’s exact test

The inflammatory markers CRP, AGP, and I-FABP were higher among cases compared to controls (*p* = 0.003, *p* = 0.024, and *p* = 0.047, respectively) (Table 2). There were no significant correlations of CRP, AGP, and I-FABP with SF or BIS in the combined sample of adolescents with and without pMDD or within the groups separately (data not shown). There was, however, a positive correlation between AGP and sTfR (rho = 0.15, *p* = 0.044) and a positive trend between CRP and sTfR (rho = 0.13, *p* = 0.073) in the combined sample of adolescents with and without pMDD. For the groups separately, there was a positive correlation between CRP and sTfR among the cases (rho = 0.31, *p* = 0.002) but not among the controls (*p* > 0.3). In addition, there was a positive correlation between AGP and sTfR among the cases (rho = 0.24, *p* = 0.017), and a positive trend between AGP and sTfR among the controls (rho = 0.18, *p* = 0.077). I-FABP did not correlated with sTfR in the combined samples of adolescents with and without pMDD (*p* = 0.5) nor within the groups separately (both *p* > 0.3).

Data from the self-reported questionnaire on the history of ID diagnosis and iron treatment are displayed in Table 3. The questionnaire was returned by 61 cases and 92 controls, which resulted in a response rate of 64% among cases and 97% among controls. Significantly more cases have ever been diagnosed with ID compared to controls (44% vs 26%, *p* = 0.020). Further, the proportion of participants receiving iron treatment at or up to 1 year before study inclusion was higher among the cases compared to the controls (28% vs 13%; *p* = 0.036). Investigating differences by sex (Supplementary Table 1), among males a significantly higher proportion of cases was ever diagnosed with ID compared to controls (7 (28%) vs 3 (7.7%), *p* = 0.039), while no such difference could be found among female participants (20 (56%) vs 21 (40%), *p* = 0.14). For iron treatment before study inclusion, a significantly higher proportion of female cases received iron treatment compared to female controls (15 (42%) vs 11 (21%), *p* = 0.033), while for male participants no such difference could be observed (2 (8%) vs 2 (5%), *p* = 0.6).

**Table 3:** Data on iron deficiency diagnosis and iron treatment in adolescents with (*n* = 61) and without (*n* = 92) paediatric major depressive disorder (pMDD) based on a self-reported questionnaire assessing this information up to 1 year before study inclusion

Characteristic	Overall	Cases	Controls	*p*-Value
Ever diagnosed with ID				**0.020**^b^
No	102 (67%)	34 (56%)	68 (74%)	
Yes	51 (33%)	27 (44%)	24 (26%)	
Treatment with iron after diagnosis of ID				> 0.9^c^
No treatment	3 (6%)	2 (7%)	1 (4%)	
Treatment with infusion/tablets/syrup/else	48 (94%)	25 (93%)	23 (96%)	
Iron treatment at or close to study inclusion^a^				**0.036**^c^
No iron treatment at or close to study inclusion	123 (80%)	44 (72%)	79 (86%)	
Iron treatment at or close to study inclusion	30 (20%)	17 (28%)	13 (14%)	

*n* (%); *p*-values in bold were statistically significant

*ID* iron deficiency

^a^Iron treatment (as oral supplements or intravenous) at or close to study inclusion defined as treatment up to 1 year before inclusion

^b^Pearson’s Chi-squared test

^c^Fisher’s exact test

A summary of the multivariate logistic regression models on the associations of iron status parameters and inflammatory markers with pMDD is displayed in Table 4. Higher sTfR concentrations were associated with lower odds for depression (OR = 0.61[0.42–0.82], *p* = 0.003). There was a trend for higher BIS being associated with higher odds for depression (OR = 1.12[0.99–1.28], *p* = 0.083). Finally, higher AGP concentrations were associated with higher odds for depression (OR = 6.10[1.11–37.4], *p* = 0.042).

**Table 4:** Multivariate logistic regression models assessing associations of different iron status and inflammation parameters with paediatric major depressive disorder (pMDD) diagnosed using the Children’s depression scale-revised (CDRS-R)

	Odds ratio (OR)	95% CI OR lower	95% CI OR upper	*p*-Value
Iron status parameters				
SF	1.01	1.00	1.02	0.2
sTfR	0.61	0.42	0.82	**0.003**
BIS	1.12	0.99	1.28	0.083
Inflammation parameters				
CRP	0.96	0.79	1.11	0.6
AGP	6.10	1.11	37.4	**0.042**
I-FABP	1.00	1.00	1.00	0.9

*p*-values in bold were statistically significant

*BIS* body iron stores; *SF* serum ferritin; *sTfR* soluble transferrin receptor; *AGP* alpha-1-acid-glycoprotein; *CRP* C-reactive protein; *I-FABP* intestinal fatty acid binding protein; *CI* confidence interval; *OR* odds ratio

The dependent variable was the diagnosis of depression (CDRS-R ≥ 40). The independent variables were iron status indicators or inflammation parameters. Models with iron status parameters were controlled for sex, age, education level, BMI-for-age *z*-scores, antidepressant use, and AGP. Models with inflammation parameters were controlled for sex, age, education level, BMI-for-age *z*-scores, and antidepressant use

## Discussion

This controlled, matched case–control study in Swiss adolescents with and without pMDD did not confirm our hypothesis of depressed adolescents having a lower iron status than their healthy counterparts. While there was no difference in SF concentrations and BIS between cases and controls, cases had significantly lower sTfR concentrations compared to controls, indicating that adolescents with pMDD have a better iron status. We found significantly higher concentrations of the inflammatory markers CRP and AGP, as well as the marker of intestinal permeability I-FABP in cases compared to controls, confirming our hypothesis of inflammation and impaired gut permeability being more prevalent among adolescents with pMDD compared to controls. However, only AGP correlated positively with sTfR concentrations (higher sTfR being indicative of lower iron status). Compared to the controls, a significantly higher proportion of cases was ever diagnosed with ID and was receiving iron treatment up to 1 year before study inclusion. Analysis by sex revealed that it was the female cases who were more likely to have received iron treatment prior to study inclusion compared to the female controls. This may explain the better iron status observed in cases compared to controls.

Previous studies have found low iron status to be associated with depression [26–28, 54], but overall, the evidence is limited and ambiguous [55, 56]. For instance, lower SF concentrations have been associated with depression in adult Iranian students [26], in English elderly persons (> 65 years of age) [27], and in middle-aged male Japanese workers [28]. On the other hand, lower SF concentrations were not observed in depressed versus non-depressed elderly people (65–83 years) in Germany [55] or in Chinese women after giving birth [56]. For adolescents specifically, there are limited and inconsistent data on the associations between iron status parameters and depressive disorders. In Mexican adolescents, female participants (*n* = 403) aged 12–20 years with ID had greater odds for being “likely depressed” (OR = 2.01) or “highly likely depressed” (OR = 2.80) [54]. In New Zealand, higher body iron stores in males were associated with greater depressive symptoms while this could not be shown in females [57]. On the other hand, adolescents with heavy menstrual bleeding and ID or IDA in Michigan, US, were not more likely to be depressed in comparison with adolescents without ID or IDA [58]. Similarly, no associations were observed between iron status and internalising problems (including depression) in 6-year-old Spanish children [59]. In this case–control study, we found significantly lower sTfR concentrations in cases compared to controls, in the entire group and in both males and females separately. Elevated sTfR concentrations in the absence of other conditions causing erythroid hyperplasia usually indicate ID erythropoiesis (IDE) [60]. In addition, there was a trend for higher BIS among the cases compared to the controls and higher odds for depression with higher BIS. Therefore, against our hypothesis, our results point towards a better iron status among cases compared to controls. Furthermore, we found no difference in iron status based on SF concentrations, with a prevalence of low iron stores (defined as adjusted SF < 15 µg/L) of 19% in cases and 18% in controls. However, the better iron status in depressed adolescents compared to healthy controls based on sTfR and BIS, and the lack of difference between cases and controls based on SF might be attributed to the differences in ID diagnosis history and treatment.

ID and depression can manifest in similar ways such as fatigue, poor school performance, decreased cognitive abilities, and behavioural problems [14, 15]. We, therefore, investigated the history of ID diagnosis as well as iron treatment prior to and at study inclusion. Indeed, the prevalence of ever being diagnosed with ID was significantly higher among cases (44%) compared to controls (26%). Furthermore, we found that 28% of adolescents with pMDD were treated with iron at or close to study inclusion compared to 14% of healthy controls. Effects of iron treatment are detectable up to 1 year after administration [45]. Thus, even though the response rate to the questionnaire was only 64% in cases compared to 97% in controls, these findings indicate that ID in cases may have been masked by recent or ongoing iron treatment and may also explain the better iron status based on sTfR. The prevalence of ID was higher in female adolescents than males 26% vs 11%, which is expected as female adolescents have higher iron requirements than males to compensate for menstrual iron losses. Consequently, the proportion of adolescents with a diagnosis of ID and iron treatment was also higher among females than males, and the difference in iron treatment between cases and controls was driven by the female participants. It remains to be speculated whether the higher prevalence of ID in females may contribute towards the higher risk of depression observed in females. Thus, future research investigating associations between iron status and depression should assess and control for iron treatment up to 1 year before data assessment, especially in adolescent females and women of reproductive age who are at increased risk of developing ID and, therefore, more likely to have a history of ID diagnosis and iron treatment.

There are two ways in which depression-associated inflammation was of interest in the current analysis. On one hand, biomarkers of iron status, particularly SF, are prone to bias due to inflammation. SF is an acute phase protein, and therefore its concentration is elevated in response to inflammation [61], which may mask ID. On the other hand, inflammation can decrease intestinal iron absorption, mediated by an upregulation of hepcidin, and therefore, increase the risk for ID. We have thus determined the inflammatory markers CRP, AGP as well as I-FABP (marker of intestinal permeability) to better understand the interrelations between iron status, depression, and inflammation. Our results support the inflammatory theory of depression [9]. Although the cases did not show clinically significant signs of infection, their CRP and AGP concentrations were significantly elevated in comparison to those of controls, indicating a state of subclinical systemic inflammation. In addition, higher AGP concentrations were associated with higher odds for pMDD. However, this increased inflammatory state did not seem to influence SF or BIS measures in a significant way, as these parameters did not correlate with CRP, AGP or I-FABP. In contrast, we found positive correlations of AGP and CRP with sTfR. In addition to the markers of systemic inflammation, also I-FABP, an indicator of intestinal permeability, was significantly higher among the cases compared to the controls, indicating reduced gut integrity. This is in line with previous studies that found elevated I-FABP concentrations among Spanish adult MDD patients [62] and adult MDD patients in the US [63] compared to healthy counterparts. Thus, taken together, our data might support the evidence for fragilisation of the gut barrier [64].

Our study has several strengths and limitations. An important strength of this study is our thoroughly matched case–control study design. In addition, the inclusion of the cases was based on a clinical diagnosis of pMDD of moderate to severe depressive symptoms. Furthermore, we assessed the severity of the depressive symptoms with a comprehensive standardised interviewer-administered assessment which included non-verbal items. However, despite the strengths of the study design, due to its observational nature, it does not allow to draw causal conclusions. A major limitation of this study is the limited response rate to the questionnaire on the history of ID diagnosis and iron treatment among the cases. This can be explained by the way the data were collected: Our questionnaire on the history of ID diagnosis and treatment was not part of the original Omega-3 pMDD intervention trial and was only introduced retrospectively for a large proportion of the cases as well as some of the controls. Some of the cases were asked to complete the questionnaire up to four years after inclusion into the study, which led to a low response rate and potential misreporting. Nevertheless, we found cases to be significantly more likely to receive iron treatment at study inclusion compared to healthy controls. However, we were not able to determine whether previous iron treatment was a confounder in our models determining the associations between iron status and depression. In addition, we did only consider iron supplements and IV iron treatment in the analysis, while some of the participants did consume other supplements (e.g. multivitamin/mineral supplements) that may have contained iron but were not precisely specified.

To conclude, our findings do not support the hypothesis of depression being associated with lower iron status. However, as iron supplementation was more prevalent among cases compared to controls, we cannot rule out that this may have influenced our findings. On the other hand, our data confirm the inflammatory theory of depression, showing both increased systemic inflammation and intestinal permeability among cases compared to controls. Future studies investigating the associations between iron status and depression should consider the history of iron treatment as a potential confounding factor in depressed individuals, particularly in females. Meanwhile, in clinical practice, depression should be considered alongside ID in children and adolescents with symptoms of fatigue, poor school performance and decreased cognitive performance.

### Supplementary Information

Below is the link to the electronic supplementary material.

Supplementary file1 (DOCX 272 KB)

## Data Availability

Dataset analyzed for this study is available from the corresponding author upon reasonable request.
