# Supplementary material for: Iron status in Swiss adolescents with paediatric major depressive disorder and healthy controls: a matched case–control study
Source: Eur J Nutr. 2024 Jan 24;63(3):951–63. doi: 10.1007/s00394-023-03313-7 (PMC10948461; doi:10.1007/s00394-023-03313-7)
Supplement: Supplementary file 1 — Supplementary file1 (DOCX 272 KB) [file 394_2023_3313_MOESM1_ESM.docx]

## Supplementary Table 1: Summary of iron status parameters as well as data on iron deficiency diagnosis and iron treatment for adolescents with and without paediatric major depressive disorder (pMDD)

|  | Female | | | | Male | | | |
| --- | --- | --- | --- | --- | --- | --- | --- | --- |
| Characteristic | N | Cases | Controls | p-value | N | Cases | Controls | p-value |
| SF [μg/L] | 55/55 | 31.8 (17.6, 46.9) | 29.2 (16.3, 43.6) | 0.7^2^ | 40/40 | 74.2 (33.8, 64.4) | 42.1 (26.0, 53.2) | 0.3^2^ |
| sTfR [mg/L] | 55/55 | 4.44 (4.01, 5.73) | 5.20 (4.75, 6.10) | **0.006^2^** | 40/40 | 4.50 (4.00, 5.10) | 5.40 (4.78, 6.10) | **<0.001^2^** |
| Iron deficiency (ID)^Δ^ | 55/55 | 14 (25%) | 14 (25%) | >0.9^3^ | 40/40 | 5 (13%) | 4 (10%) | >0.9^3^ |
| Ever diagnosed with ID | 36/53 | 20 (56%) | 21 (40%) | 0.14^3^ | 25/39 | 7 (28%) | 3 (7.7%) | **0.039^3^** |
| Iron treatment at or close to study inclusion^*^ | 36/53 | 15 (42%) | 11 (21%) | **0.033^4^** | 25/39 | 2 (8%) | 2 (5%) | 0.6^4^ |
| Median (Interquartile range); n (%); p-values in bold were statistically significant  AGP: alpha-1-acid-glycoprotein; CRP: C-reactive protein; I-FABP: Intestinal fatty acid binding protein; SD: standard deviation; SF: Serum ferritin; sTfR: soluble transferrin receptor | | | | | | | | |
| ^Δ^ID was defined as adjusted SF <15 μg/L and/or elevated sTfR >8.3 mg/L  ^*^Iron treatment (as oral supplements or intravenous) at or close to study inclusion defined as treatment up to 1 year before inclusion.  ^2^Wilcoxon rank sum test; ^3^Pearson's Chi-squared test; ^4^Fisher's exact test | | | | | | | | |
